# Isolation, Characterization, and Biocompatibility of Bisporitin, a Ribotoxin-like Protein from White Button Mushroom (*Agaricus bisporus*)

**DOI:** 10.3390/biom13020237

**Published:** 2023-01-26

**Authors:** Sara Ragucci, Hafiza Zumra Fatima Hussain, Andrea Bosso, Nicola Landi, Angela Clemente, Paolo Vincenzo Pedone, Elio Pizzo, Antimo Di Maro

**Affiliations:** 1 Department of Environmental, Biological and Pharmaceutical Sciences and Technologies (DiSTABiF), University of Campania ‘Luigi Vanvitelli’, Via Vivaldi 43, 81100 Caserta, Italy; 2 Department of Biology, University of Naples ‘Federico II’, Via Cinthia 26, 80126 Naples, Italy; 3 Centro Servizi Metrologici e Tecnologici Avanzati (CeSMA), University of Naples ‘Federico II’, 80126 Naples, Italy

**Keywords:** α-fragment, champignon, edible mushrooms, ribonucleases, protein purification

## Abstract

White button mushroom (*Agaricus bisporus* (J.E. Lange) Imbach) is one of the widely consumed edible mushrooms. Indeed, *A. bisporus* fruiting bodies are a rich source of nutrients and bioactive molecules. In addition, several enzymes with biotechnological applications are found in *A. bisporus* (e.g., enzymes for lignocellulose degradation). Here, a novel ribotoxin-like protein (RL-P) from the edible mushroom *A. bisporus* was purified and characterized. This RL-P, named bisporitin, is a monomeric protein (17-kDa) exhibiting specific ribonucleolytic activity by releasing the α-fragment (hallmark of RL-Ps) when incubated with rabbit ribosomes. In addition, bisporitin shows magnesium-dependent endonuclease activity and displays a similar far-UV CD spectrum as ageritin, the prototype of RL-Ps, isolated from *Cyclocybe aegerita* fruiting bodies. Interestingly, bisporitin is the first member of RL-Ps to have noticeably lower thermal stability (T_m_ = 48.59 ± 0.98 °C) compared to RL-Ps isolated in other mushrooms (T_m_ > 70 °C). Finally, this protein is only partially hydrolyzed in an in vitro digestive system and does not produce adverse growing effects on eukaryotic cell lines. This evidence paves the way for future investigations on possible bioactivities of this RL-P in the digestive system.

## 1. Introduction

White button mushroom (*Agaricus bisporus* (J.E. Lange) Imbach), also known as champignon, is an edible basidiomycete mushroom belonging to Agaricales order [1] and is one of the most widely cultivated in the world, representing 40% of mushrooms produced for human consumption [2]. In Europe, it has been cultivated since ancient Romans and nowadays is widely consumed due to its high nutritional values as well as the presence of different bioactive compounds contributing to the maintenance of good human health [3,4,5]. Indeed, *A. bisporus* is a source of bioactive molecules suitable for several therapeutical applications [6,7], such as anticancer, antioxidant, and immunomodulatory applications [4,7,8]. In addition, its fruiting bodies are notable natural sources of lovastatin, an inhibitor of HMG-CoA reductase, an approved-to-market drug used in the treatment of hypercholesterolemia [9].

In addition, several bioactive proteins or enzymes with biotechnological interest were retrieved in *A. bisporus* fruiting bodies. Among them, lectins are abundantly synthesized storage proteins that exhibit interesting biomedical properties, such as immunomodulatory and anticancer activities [10,11], or are even able to inhibit the reverse transcriptase of HIV1 [12]. In addition, other well-characterized enzymes from *A. bisporus* are: (i) laccases implicated in fruiting body formation, pigmentation, lignin degradation, and stress defense [13]; (ii) acid phosphatases involved in the synthesis of mannitol [14]; (iii) galactosidases, displaying hydrolytic efficacy toward raffinose family oligosaccharides [15]; and (iv) lignin-degrading peroxidases, displaying a role in lignin degradation by wood-rotting fungi [16].

Intriguingly, toxic proteins with the ribosome as a target, known as ribotoxin-like proteins (RL-Ps), have been recently identified in other edible cultivated mushrooms belonging to basidiomycetes (i.e., *Cyclocybe aegerita* [17], *Boletus edulis* [18], *Pleurotus eryngii* [19] and *Pleurotus ostreatus* [20]). RL-Ps are enzymes able to inhibit protein synthesis, analogs to ribotoxins isolated from ascomycetes fungi [21,22], and may have toxic effects on human health. The presence of such toxic proteins in *A. bisporus* is, to date, still unknown and is an interesting issue since *A. bisporus* fruiting bodies are also consumed as raw food (e.g., in salads).

RL-Ps are specific ribonucleases found in the fruiting bodies of edible mushrooms belonging to the Basidiomycota division [23]. These enzymes cleave a single phosphodiester bond located within the universally conserved Sarcin-Ricin Loop (SRL) of 23–28S rRNAs damaging the ribosomes [17,24]. The loss of ribosome integrity prevents protein biosynthesis, leading to cell death by apoptosis [18,25,26]. RL-Ps are small (~140 amino acid residues: ~16-kDa) and basic proteins with high thermal stability (T_m_ > 70 °C) [23]. In addition, ageritin, the prototype of this novel group of specific ribonucleases, isolated from *Cyclocybe aegerita* fruiting bodies, exhibits antifungal activity against the green mold *Penicillium digitatum* [26] and has cytotoxic effect towards insect Sf9 cell lines [19].

Finally, most of these enzymes exhibit cytotoxic effects toward several malignant cell lines [18,23,27]. Considering as above, it is reasonable to hypothesize that these specific ribonucleases are involved in basidiomycetes defense mechanisms, although the biological role of RL-Ps needs to be investigated.

Overall, RL-Ps are enzymes of interest, considering their structural and biological features. Indeed, most of the family members can be novel candidates for biotechnological applications. In particular, RL-Ps could be used for the production of bioconjugates with selective cytotoxic action (e.g., immunotoxins) [28] in cancer therapy or as biopesticides in agriculture to improve crop resistance [26].

In this pilot study, we preliminarily screened the putative presence of RL-P members in *A. bisporus* by analyzing the capability of the protein extract to inhibit ribosomal protein synthesis. Thus, we proceeded to isolate for the first time from *A. bisporus* the protein responsible for this property, here named bisporitin. Further, a first overview of enzymatic, structural, and biological features of bisporitin has been reported. Collected data highlighted peculiar structural and biological properties of this novel RL-P as its unusually low T_m_ and negligible cytotoxicity on eukaryotic cells.

## 2. Materials and Methods

### 2.1. Reagents

Most of the reagents were obtained from Merk Life Science s.r.l., Milano, Italy. Resins for chromatography were obtained from Cytiva, Buccinasco (MI), Italy, as previously reported [18,19]. Reagents for Sodium Dodecyl Sulphate-PolyAcrylamide Gel Electrophoresis (SDS-PAGE) were obtained from Bio-Rad S.r.l. Rome, Italy. Single-stranded salmon sperm DNA and nuclease-treated rabbit reticulocyte lysate system were purchased from Sigma-Aldrich (St. Louis, MO, USA) and from Promega (Madison, WI, USA), respectively. All other reagents and chemicals were of analytical grade.

The following buffers have been used: buffer A: 5 mM Na-phosphate, pH 7.2, containing 0.14 M NaCl; buffer B: 10 mM Na-acetate, pH 4.0 and buffer C: 5 mM Na-phosphate, pH 7.2.

### 2.2. Mushroom Samples for Bisporitin Purification

*Agaricus bisporus* (J.E. Lange) Imbach fruiting bodies were purchased in local markets (Campania Region; Caserta, Italy). Edible parts (pileus, hymenium, and stipe) were washed and stored at −80 °C until further analyses.

### 2.3. Protein Purification

Bisporitin was purified from *A. bisporus* fruiting bodies using a general protocol for the purification of basic proteins [17,18,19]. Fruiting bodies (300 g) were homogenized in 1.0 L buffer A by 20 s bursts at full power using a Waring Blendor (Waring Products, Torrington, CT, USA). The homogenate was stirred for 12 h at 4 °C, filtered through Miracloth filter paper (Merk Life Science S.r.l, Milan, Italy), and centrifuged at 15,000× *g*, 4 °C for 1 h (Avanti J, Beckman Coulter S.r.l., Milan, Italy). The crude extract was adjusted to pH 4.0 with glacial acetic acid and then centrifuged at 15,000× *g*, 4 °C for 1 h. The supernatant was loaded onto a column (5 × 15 cm) containing Streamline™ SP (Cytiva, Milan, Italy) equilibrated in buffer B at a 3.0 mL/min flow rate. After sample loading, the resin was washed in buffer B and then in buffer C until the A_280_ nm was below 0.01 OD. Bound basic proteins were eluted with 1M NaCl in buffer C, monitoring the absorbance of the eluate at 280 nm. Fractions (10 mL) were pooled and concentrated in an Amicon cell concentrator (MWCO 10 kDa; Merk Life Science, Milan, Italy). The insoluble material was removed by centrifugation at 15,000× *g* for 20 min at 4 °C. Subsequently, the protein pool able to inhibit protein synthesis in vitro was subjected to gel filtration by Hi-Load 26/60 Superdex 75 prep grade (Cytiva, Bucinasco (MI), Italy) on an AKTA Purifier System (Amersham Pharmacia; Milan, Italy). After size-exclusion chromatography (equilibrated and eluted with 0.30 M NaCl in buffer C; flow rate 2.5 mL min^−1^), fractions able to inhibit protein synthesis in vitro were pooled, dialyzed against buffer C and further purified by cation exchange chromatography on an S-Sepharose fast flow column (20 × 1.6 cm), equilibrated in buffer C and eluted with a NaCl gradient up to 0.15 M (buffer C, 500 mL; buffer C containing NaCl, 500 mL; total volume 1 L, flow rate 0.60 mL min^−1^) using a peristaltic pump. In order to pool bisporitin, the fractions were assayed for ribotoxin activity (inhibition of protein synthesis or release of the α-fragment) as previously reported [17,29]. Purified ribotoxin-like protein was pooled, dialyzed against water, freeze-dried, and stored at −20 °C until use.

### 2.4. Analytical Procedures

All general methodologies used for the analytical characterization of bisporitin, such as SDS-PAGE and determination of protein concentration by colorimetric assay (BCA assay; Promega, Madison, WI, USA), were carried out as described elsewhere [19].

### 2.5. Protein Synthesis Inhibition In Vitro

Inhibition of protein synthesis in a lysate of rabbit reticulocytes through a nonradioactive method based on a combined transcription/translation system was performed as described elsewhere [29,30].

### 2.6. Enzymatic Assays

#### 2.6.1. Ribonuclease Activity on Yeast High-Molecular-Weight RNA

Ribonuclease activity was assayed by monitoring the shift in the maximum absorbance of methylene blue upon intercalation into yeast high-molecular-weight RNA at 688 nm [31]. As a reference enzyme, ribonuclease A from the bovine pancreas was used. Results are mean values of two experiments performed in triplicate.

#### 2.6.2. Ribonucleolytic Activity on Rabbit Ribosomes

The specific activity of ribotoxins, manifested by the release of α-fragment from rRNA after enzymatic action on ribosomes, was detected as described elsewhere [17,32]. Briefly, the enzymes were incubated with ribosomes; then, total proteins were removed, and RNA was incubated with or without acid aniline. Finally, RNA was precipitated to remove aniline, subjected to electrophoresis in a 7 M urea/5% (*w*/*v*) polyacrylamide gel, and stained with ethidium bromide.

#### 2.6.3. Nicking Endonuclease Activity on Supercoiled pUC18 DNA

Nicking activity experiments were performed as previously reported [26]. Each reaction contained 2.5 μg of protein and 200 ng of pUC18 DNA in 10 mM Tris-Cl, 50 mM NaCl and 50 mM KCl, pH 7.8 with or without 5.0 mM MgCl_2_ or 25 mM EDTA (final volume 10 μL). Samples were incubated for 1 h at 37 °C, run on agarose gel (0.8%) in TAE buffer (0.04 M Tris, 0.04 M acetate, 1.0 mM EDTA, pH 8.0), and visualized by ethidium bromide staining (0.50 μg/mL). HindIII linearization was obtained by incubating 250 ng of pUC18 with 1.5 units of HindIII (Amersham Life Sciences Inc., Arlington, MA, USA) according to manufacturer instructions.

### 2.7. Circular Dichroism and Thermal Stability Determination

CD spectrum of bisporitin in the far UV region was acquired by Jasco J-815 dichrograph (Jasco Europe, Lecco, Italy) at room temperature (protein concentration 0.16 mg per mL in 10 mM Na-phosphate, pH 7.2) as described elsewhere [19].

Temperature melting (T_m_) of bisporitin was obtained by heat-induced denaturation. Signals were monitored at 278 nm on a UV-VIS Cary 100 spectrometer (Agilent Technologies Italia S.p.A., Milan, Italy), equipped with a Peltier temperature controller. The protein (0.16 mg per mL) in 10 mM Na-phosphate, pH 7.2, was subjected to an increase of 1.0 °C per minute (range of 20–95 °C). T_m_ of the unfolding curve was determined by fitting data to the Boltzmann model using Prism 8 software (GraphPad Software Inc., San Diego, CA, USA).

### 2.8. In Vitro Digestibility

In vitro digestibility of bisporitin was performed as described elsewhere [33]. Briefly, protein dissolved in 0.1 M HCl was incubated with pepsin (enzyme/substrate, 1:100) for 2 h at 37 °C. Subsequently, following pepsin inactivation by NaOH, ammonium carbonate was added to a final concentration of 50 mM. Trypsin was added to this mixture (enzyme/substrate, 1:100) and incubated for 2 h at 37 °C. Finally, to inactive trypsin, each sample was boiled for 10 min. The in vitro digestibility was checked by SDS-PAGE analysis under reducing conditions [19].

### 2.9. Cell Cultures

The cytotoxic effect of bisporitin was evaluated on HaCaT and A431 cell lines, respectively, human normal and tumoral keratinocytes, as well as HeLa cells from cervical carcinoma, by performing the 3-(4,5-dimethylthiazol-2-yl)-2,5 diphenyltetrazolium bromide reduction inhibition assay (MTT assay) [34], designed to be used for the spectrophotometric quantification of cell metabolic activity. Briefly, 3 × 10^3^ cells were seeded into a 96-well plate and incubated at 37 °C in the presence of 5% CO_2_. The medium was then replaced with 100 μL of fresh medium containing bisporitin solution at a final concentration ranging from 0 to 10 μM/well. After 24, 48, and 72 h of incubation at 37 °C, the medium was removed, and 100 μL of 0.5 mg/mL MTT solution diluted in Dulbecco’s modified Eagle’s medium (DMEM) purchased from Lonza (Basel, Switzerland) without red phenol was added. After 4 h of incubation at 37 °C, the resulting insoluble formazan salts were solubilized in anhydrous isopropanol containing 40 mM HCl and quantified by measuring the absorbance at λ = 570 nm, using an automatic plate reader spectrophotometer (Synergy HTX Multi-Mode Reader-BIOTEK, Winooski, VT, USA). Cell survival was expressed as mean of the percentage values compared to control represented by untreated cells.

### 2.10. Statistical Analysis

Statistical analysis was achieved using Prism 8 software (GraphPad Software Inc., San Diego, CA, USA). Values are reported as the means ± SD of biological replicates (* *p* < 0.05, ** *p* < 0.01, *** *p* < 0.001, or **** *p* < 0.0001) compared to the respective controls (one-way ANOVA).

## 3. Results and Discussion

### 3.1. Purification of Bisporitin

**Figure 1:**
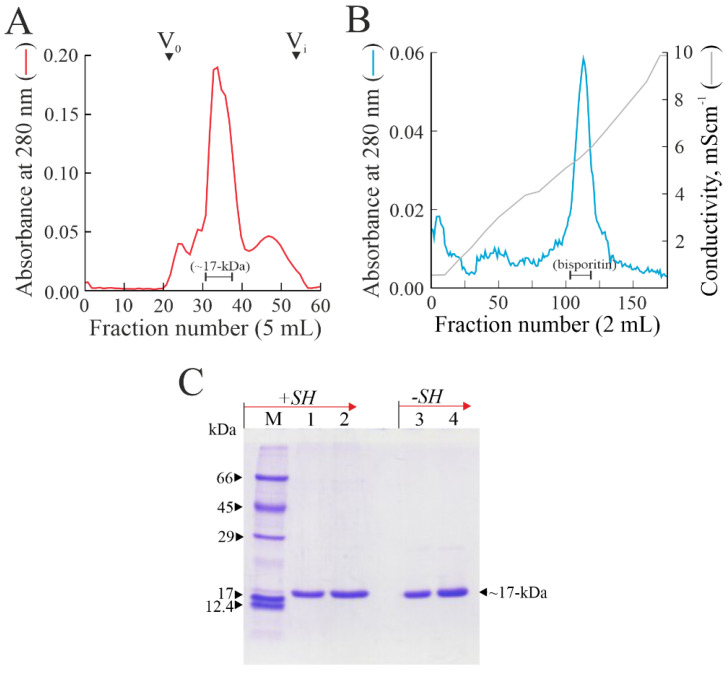
Purification of bisporitin from *A. bisporus* fruiting bodies. (**A**) Elution profile from gel-filtration in Superdex 75 column of basic protein extract after cationic chromatography in S-Streamline column (see Materials and Methods). V_0_ and V_i_ indicate the void and inclusion volume of gel-filtration column, respectively. (**B**) Elution profile from cation exchange chromatography in an S-Sepharose column of protein pool with an elution volume of ~17-kDa; (**C**) SDS-PAGE in a 12% polyacrylamide gel of bisporitin with (+SH) or without (−SH) reducing agent. Lane M, molecular markers; lanes 1–3 and 2–4, 3.0 µg and 6.0 µg of bisporitin, respectively.

Preliminarily to following purification steps, a raw extract prepared from *A. bisporus* fruiting bodies was analyzed for its inhibitory activity on protein synthesis by a rabbit reticulocyte lysate system. In order to assess whether the inhibition was due to the presence of RP-Ls, a purification procedure was performed, considering the basic pI (pI ≥ 9.0) and the molecular weight (~16-kDa) of the members of this enzyme family. In particular, the acidic protein fraction obtained by saline extraction from *A. bisporus* fruiting bodies at pH 4.0 was first subjected to cationic stepwise chromatography in an S-Streamline column. Then, protein fractions eluted with NaCl at pH 7.2 were pooled (basic protein pool) and separated by gel-filtration chromatography on a Sephadex G-75 column. The inhibitory activity of this chromatographic step was fractionated into a single protein peak with an elution volume of ~17-kDa (Figure 1A).

Eluted fractions of this peak, containing non-homogenous proteins, were pooled and subjected to a further cationic exchange chromatographic step in a S-Sepharose column. As shown in Figure 1B, a principal protein peak was eluted. Fractions of this protein peak from 105 to 120 contained a homogenous protein with an electrophoretic migration of 17-kDa, named bisporitin (Figure 1B). Afterward, these fractions were pooled, dialyzed, and concentrated. The yield as average of five different purification procedures was 0.91 ± 0.41 mg of bisporitin per 1.0 kg of fresh *A. bisporus* fruiting bodies.

Finally, the electropherogram of pure protein obtained by SDS-PAGE with or without reducing conditions showed that bisporitin consists of a single polypeptide chain with a molecular weight of ~17-kDa (Figure 1C).

### 3.2. Enzymatic Properties of Bisporitin

**Figure 2:**
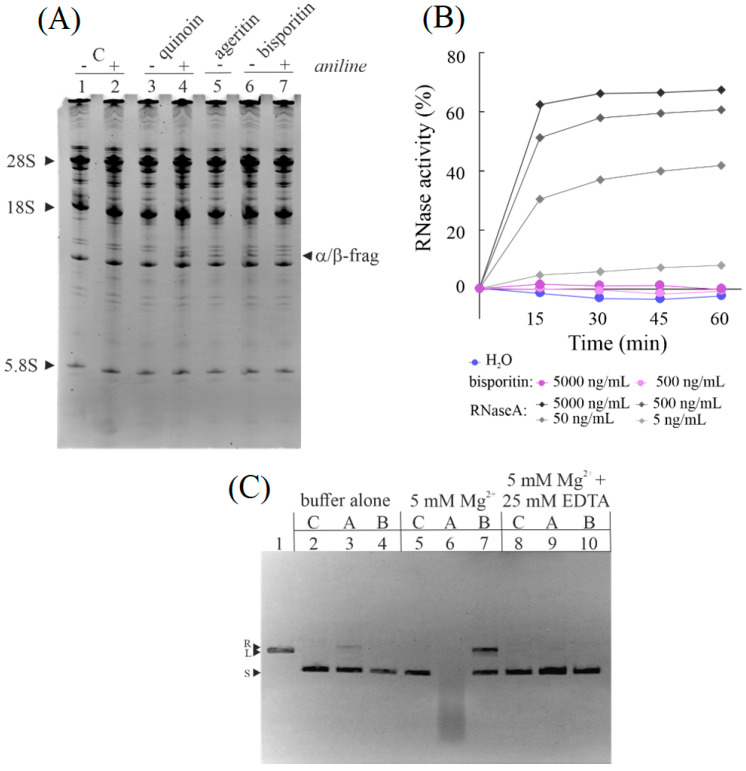
Enzymatic action of bisporitin on different substrates. (**A**) Activity on rabbit ribosomes. Release of α-fragment from bisporitin in the absence (lane 6) or presence (lane 7) of aniline treatment. Ribonucleolytic action of RL-P ageritin (α-fragment released without aniline treatment, lane 5) and rRNA N-glycosylase activity of type 1 RIP quinoin (β-fragment released after aniline treatment, lane 4) used as reference. C, untreated control, lanes 1 and 2 in the absence or presence of aniline treatment. Each lane contained 3.0 μg of RNA isolated from untreated (control) ribosomes or treated with the toxin. The arrows indicate the RNA fragment (Endo’s fragment) released as a result of the action of enzymes. Markers 28S, 18S and 5.8S are indicated. (**B**) Ribonuclease activity of bisporitin compared with ribonuclease A from bovine pancreas. Yeast RNA was incubated with the protein concentrations indicated in the figure, as described in Materials and Methods, and RNA degradation was estimated by monitoring the decrease in absorbance at 688 nm. (**C**) Nicking endonuclease activity of bisporitin on pUC18 DNA. In total, 200 ng of plasmid DNA was incubated with ageritin (A, 2.5 μg) or bisporitin (B, 2.5 μg) in buffer alone (lanes 3 and 4, respectively); with 5 mM Mg^2+^ (lanes 6 and 7, respectively) or 25 mM EDTA in presence of 5.0 mM Mg^2+^ (lanes 9 and 10, respectively). Lane 1, pUC18 treated with HindIII; lanes 2, 5, and 8, pUC18 DNA control in buffer alone, with Mg^2+^ or 25 mM EDTA in presence of 5.0 mM Mg^2+^, respectively. R, L, and S: relaxed, linear, and supercoiled forms of pUC18, respectively; 0.8% agarose gel electrophoresis.

RL-Ps are specific ribonucleases from edible mushrooms able to damage ribosomes cleaving a single phosphodiester bond in the SRL region. This enzymatic action prevents ribosome interaction with elongation factor EF-2, blocking protein synthesis. The hallmark of this enzymatic action is the release of a specific diagnostic RNA fragment at the 3′ end of 28S rRNA, known as α-fragment [24]. Thus, to ascertain whether the ribosome inhibitory activity of *A. bisporus* raw extract was due to bisporitin, it was analyzed if the purified protein, incubated with rabbit ribosome lysates, was able to release the α-fragment. As displayed in lane 6 of Figure 2A, bisporitin releases the α-fragment even without aniline treatment, the reason for which it could be a novel type of RL-P.

Subsequently, to ascertain that bisporitin did not possess a nonspecific hydrolytic action, the protein was assayed in the presence of yeast RNA, comparing it to pancreatic RNase A. It is evident that bisporitin had no effect on yeast RNA up to 5.0 μg/mL, whereas the effect of pancreatic RNase A was evident at a concentration 100-fold lower (Figure 2B).

Finally, in previous works, we reported that some RL-Ps, such as ageritin from *C. aegerita* [26] and ostreatin from *P. ostreatus* [20], exhibit nicking endonuclease activity on plasmid DNA with or without metal ions. In this framework, we tested the bisporitin nicking endonuclease activity on the plasmid pUC18. Bisporitin promoted the conversion of supercoiled pUC18 DNA into the relaxed form in the presence of Mg^2+^ (lane 7, Figure 2C), while it did not promote such conversion in the absence of this bivalent ion. On the other hand, EDTA chelating agent reversed this activation (lane 10, Figure 2C). However, the nicking endonuclease activity of bisporitin was lower than that of ageritin, which was able to promote the conversion of supercoiled pUC18 in linear form in ‘buffer alone’ (lane 3, Figure 2C) and degradation in the presence of Mg^2+^ (lane 6, Figure 2C).

Overall, these results clearly indicate that bisporitin is a ribotoxin-like protein enzymatically susceptible to the presence of ions, despite being less active than ageritin [35].

### 3.3. Spectroscopic Properties and Thermostability of Bisporitin

**Figure 3:**
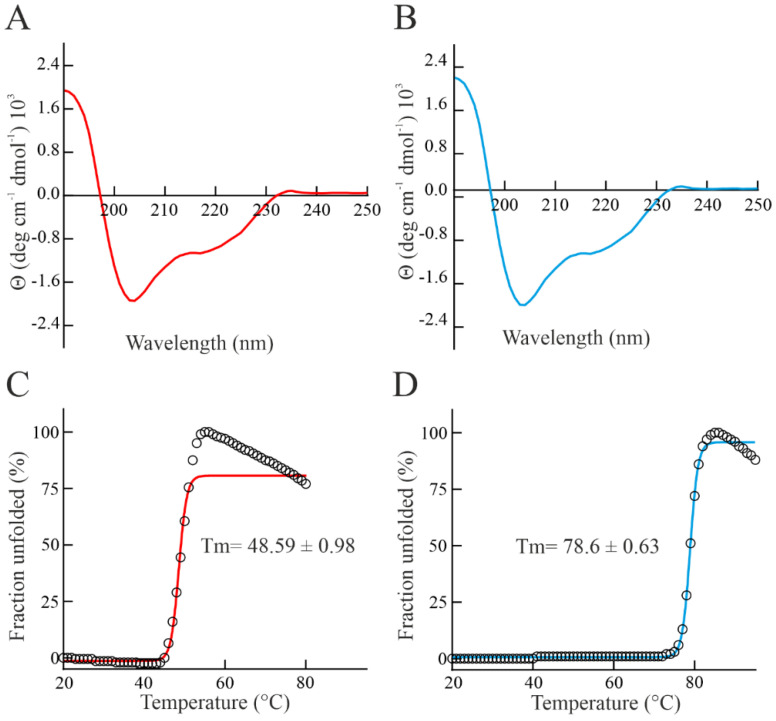
Spectroscopic properties and thermostability of bisporitin compared to ageritin. (**A**,**B**) far-UV CD spectra of bisporitin (red line) and ageritin (blue line), respectively. Spectra obtained as described in Materials and Methods were acquired at 20 °C; [protein] = 0.16 mg mL^−1^). (**C**,**D**) thermal denaturation curves of bisporitin (red line) and ageritin (blue line), respectively. Fraction unfolded at 278 nm is plotted as a function of temperature; [protein] = 0.16 mg mL^−1^.

In order to preliminary investigate the structural features of bisporitin, a study by Circular Dichroism spectroscopy (CD spectroscopy) at 20 °C and pH 7.2 was carried out (Figure 3A). Computational analyses of bisporitin CD spectrum highlight the presence of α- and β-elements, suggesting a typical well-structured alpha-beta fold as also retrieved for ageritin (prototype of RL-Ps, isolated from *C. aegerita* used as reference protein, Figure 3B and [35]) and other isolated enzymes belonging to this family [18,19].

A further peculiar feature of RL-Ps from edible mushrooms is the higher thermostability. Considering this, the thermal denaturation curve of bisporitin was achieved using UV-spectroscopy by measuring the increment of absorbance at 278 nm, increasing the temperature from 20 °C to 80 °C. The melting temperature (T_m_) of bisporitin was 48.59 ± 0.98 °C, Figure 3C. The thermal unfolding curve at pH 7.2 shows that this RL-P is not a highly stable protein. In particular, the T_m_ value of bisporitin is lower than that of both ageritin (78.6 ± 0.63 °C, Figure 3D and [35]) and eryngitin 3 (89.7 °C [19]) isolated from *C. aegerita* and *Pleurotus eryngii*, respectively.

### 3.4. Digestibility of Bisporitin In Vitro

Considering that white button mushrooms are also eaten as raw food, an in vitro study on the digestibility of bisporitin was carried out. Indeed, the acid conditions of the stomach and the hydrolytic effect of pepsin followed by trypsin action in the intestine can mitigate or eliminate possible toxic or inflammatory effects of proteins [36], such as RL-Ps [23,24].

**Figure 4:**
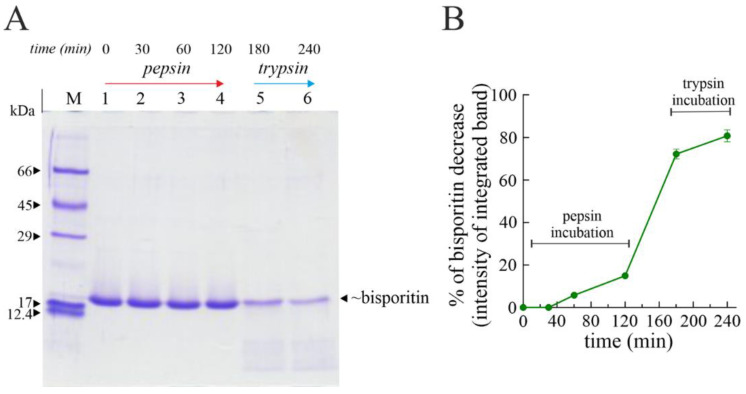
(**A**) SDS-PAGE profile of bisporitin subjected to in vitro protein digestibility. Pepsin treatment at 0, 30, 60, and 120 min (lanes 1, 2, 3, and 4, respectively) and subsequent trypsin treatment after 60 or 120 min of pre-treatment with pepsin (lanes 5 and 6, respectively). M, molecular weight markers. SDS-PAGE was carried out in 15% polyacrylamide separating gel under reducing conditions. For more details, see Materials and Methods. (**B**) Densitometric analysis on SDS-PAGE of bisporitin sampling at different times during pepsin-trypsin digestion.

Thus, we treated bisporitin with the common digestive pepsin-trypsin at different times, and the effect was evaluated by SDS-PAGE [33,37]. Pepsin-trypsin digestion of bisporitin displays that part of the toxin is slightly hydrolyzed by pepsin in acid conditions (Figure 4).

Indeed, after 120 min of proteolytic treatment with pepsin, we observed a decrease of ~15% (lane 4 Figure 4A), while only after pre-treatment with pepsin followed by trypsin treated at neutral pH for 120 min, bisporitin is mostly hydrolyzed in peptides (lane 6 in Figure 4A), showing a decrease of ~80% (Figure 4B). Overall, this experiment displays that in vivo, part of the intact protein could overcome the acidic and hydrolytic conditions of the stomach, reaching the small intestine.

### 3.5. Susceptibility of Human Cell Lines to Bisporitin

**Figure 5:**
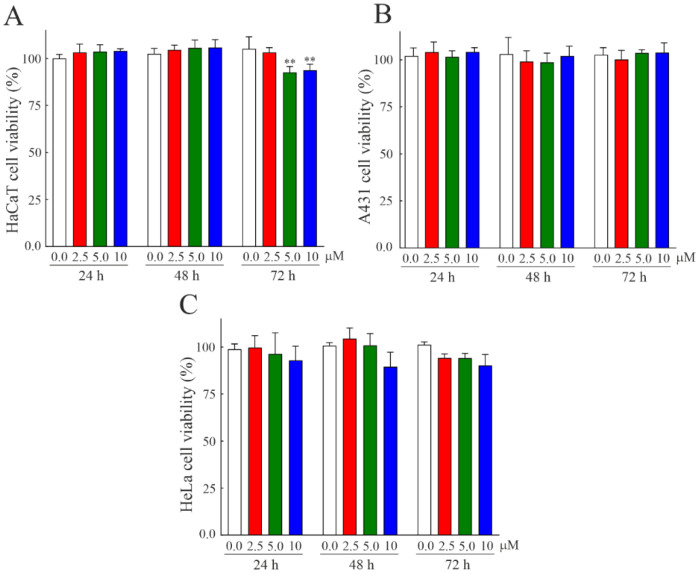
Susceptibility to cell lines of bisporitin. (**A**–**C**) HaCaT, A431, and HeLa cells incubated with different concentrations of toxin for 24, 48, or 72 h. Cell viability was evaluated by a colorimetric assay as indicated in Materials and Methods. Values of three experiments are reported as the means ± SD of biological replicates (** *p* < 0.01) compared to the respective controls (one-way ANOVA).

The partial resistivity of bisporitin to pepsin in acid conditions raises the issue of the possible cytotoxic action of this toxin after passing the stomach and overcoming the action of pepsin at acid pH. In light of this, the possible toxicity or biocompatibility of bisporitin on eukaryotic cells was evaluated. Thus, we administrated increasing concentrations of bisporitin ranging from 2.5 to 10 µM on three different cell lines: two human keratinocytes cell lines, HaCaT and A431 cells, normal and tumoral cells, respectively, as well as HeLa cells derived from cervical carcinoma. Cell metabolic activity was evaluated by performing an MTT assay after 24, 48, and 72 h from the protein administration, and as displayed in Figure 5, no significant cytotoxic effect of bisporitin was highlighted on each cell line even at the higher concentration or time, indicating the promising biocompatibility of this novel RL-P.

## 4. Conclusions

The characterization of bisporitin from *A. bisporus* fruiting bodies reported above confirms the presence of RL-Ps in different edible mushrooms. This group of enzymes is possibly involved in physiological and/or defense mechanisms, guaranteeing mushrooms homeostasis.

Bisporitin is a monomeric protein (~17-kDa) exhibiting similar CD spectroscopic features with respect to ageritin, the prototype of RL-Ps, while T_m_ value (48.59 ± 0.98 °C) is lower than other known RL-Ps, which show an average T_m_ > 70 °C. On the other hand, this enzyme displays Mg^2+^-dependent nicking endonuclease activity on plasmid DNA, as previously reported for other RL-Ps. In addition, bisporitin is slightly hydrolyzed by pepsin at pH 2.0 in an in vitro digestibility system, for which this enzyme likely overcomes the acid and hydrolytic stomach conditions without exhibiting cytotoxic effects towards tested cell lines.

Overall, further studies will be conducted to achieve the structural characterization (e.g., amino acid sequence and ions binding) to find the structural determinants that make bisporitin less toxic and stable with respect to the other known RL-Ps.

## Data Availability

The data presented in this study are available in this article.
